# Moving closer to experimental level materials property prediction using AI

**DOI:** 10.1038/s41598-022-15816-0

**Published:** 2022-07-13

**Authors:** Dipendra Jha, Vishu Gupta, Wei-keng Liao, Alok Choudhary, Ankit Agrawal

**Affiliations:** Department of Electrical and Computer Engineering, Northwestern University, Evanston, IL 60208 USA

**Keywords:** Chemistry, Materials science

## Abstract

While experiments and DFT-computations have been the primary means for understanding the chemical and physical properties of crystalline materials, experiments are expensive and DFT-computations are time-consuming and have significant discrepancies against experiments. Currently, predictive modeling based on DFT-computations have provided a rapid screening method for materials candidates for further DFT-computations and experiments; however, such models inherit the large discrepancies from the DFT-based training data. Here, we demonstrate how AI can be leveraged together with DFT to compute materials properties more accurately than DFT itself by focusing on the critical materials science task of predicting “formation energy of a material given its structure and composition”. On an experimental hold-out test set containing 137 entries, AI can predict formation energy from materials structure and composition with a mean absolute error (MAE) of 0.064 eV/atom; comparing this against DFT-computations, we find that AI can significantly outperform DFT computations for the same task (discrepancies of $>0.076$ eV/atom) for the first time.

## Introduction

Experiments and Density Functional Theory (DFT) based computations have been the primary means to know and understand the chemical and physical properties of crystalline materials^1–10^. While experiments are the only way to know the ground-truth, they are expensive and time-consuming. DFT computations offer a less expensive means for computing the electronic-scale properties of crystalline solids using first principles. This has led to collections of large DFT-computed databases like the Open Quantum Materials Database (OQMD)^5,6^, the Automatic Flow of Materials Discovery Library (AFLOWLIB)^7^, the Materials Project^8,11,12^, the Joint Automated Repository for Various Integrated Simulations (JARVIS)^9,13–15^, and the Novel Materials Discovery (NoMaD)^10^. While experimental datasets containing materials properties are still scarce, current DFT-computed datasets contain properties of ~ 10^4^–10^6^ materials which are either experimentally-observed^16^ or hypothetical. However, DFT calculations are theoretically computed for temperature at 0K, while experimental formation energies are typically measured at room temperature; this results in significant discrepancy between the DFT-computed and experimentally measured formation energies^6,17^. Such discrepancy between DFT-computed and experimentally observed materials property value can be significant, especially for the materials that undergo phase transformation between 0 and 300K; these materials contain elements from Ce, Na, Li, Ti and Sn^18^. DFT databases, such as OQMD and Materials Project, reduce this systematic error by chemical potential fitting procedures for the constituent elements with phase transformations between 0 and 300K^6^. For instance, OQMD makes the corrections to the chemical potentials of these constituent elements using a least squares fitting method using experimental formation energies of compounds containing those elements and the DFT calculated total energies of the compounds. There have also been several works that aim to correct discrepancies related to DFT calculations by making adjustments to the theoretical methodologies directly^19–21^.

Despite such adjustment to reduce the systematic error in DFT-computations, they still have significant discrepancy against experimental observations. There have been multiple recent works that compare the DFT-computed property values against the experimental observations^6,17,22,23^. For instance, Kirklin et al.^6^ compared the DFT-computed formation energy with experimental measurements of 1670 materials and found that the MAE of the Materials Project to be 0.133 eV/atom and the MAE of OQMD to be 0.108 eV/atom. Another study by Jain et al.^22^ reports the MAE of the Materials Project as 0.172 eV/atom. Recently, Jha et al.^23^ compared different DFT-computed formation energies against the experimentally measured values for a set of 463 materials from Matminer (an open source materials data mining toolkit)^24^, and found the MAE in OQMD, Materials Project and JARVIS to be 0.083 eV/atom, 0.078 eV/atom and 0.095 eV/atom respectively. For compounds with constituent elements that undergo phase transformation at low temperature, Kim et al.^17^ reports an average error of around 0.1 eV/atom between DFT-computed and experimental observed values for both Materials Project and OQMD; the average uncertainty of the experimental standard formation energy was one order of magnitude lower. Note that all these existing comparisons are made by comparing the materials composition between DFT-computed datasets and experimental data by taking the lowest formation energy (most stable compound) in case of multiple entries with duplicate compositions; these studies have ignored the structure information since they were not present in the experimental data used in these studies.

Since predictive models based solely on experimental observations would have high prediction errors due to limited availability of data for representation learning (training), predictive modeling in materials science is mostly performed by training using DFT-computed datasets. The availability of large DFT-computed datasets along with advances in the field of artificial intelligence (AI) and machine learning (ML) have spurred the interest of materials scientists in building new predictive modeling systems to understand materials and predict their properties^23,25–55^. Such predictive modeling approaches have helped in accelerating the overall process of materials discovery and design by providing a rapid screening method for potential materials candidates to reduce the composition-structure space for further DFT-computations and experiments; they have been supported by government initiatives such as the Materials Genome Initiative (MGI)^56^, leading to the novel data-driven paradigm of materials informatics^57–60^. A critical predictive modeling task in materials science is predicting the formation energy of materials to determine stability of their crystal structure^23,25,34,42–44,47,61–63^. Formation energy is an extremely important materials property since it is essential to determine compound stability, generate phase diagrams, calculate reaction enthalpies and voltages, and determine many other important materials properties. While formation energy is so ubiquitous, DFT calculations allow predictions of many other properties such as band gap energy, volume, energy above convex hull, elasticity, magnetization, moment and so on; these are expensive to measure experimentally. Such predictive modeling for determining stability of materials is performed by either building composition-only based predictive models or structure-based models trained on large DFT-computed datasets. There exist several research works in building robust and accurate predictive models for formation energy given composition using DFT-computed datasets^25,34,44^. Recently Jha et al.^23^ has demonstrated how available large DFT-computed datasets and existing experimental observations can be leveraged together using deep transfer learning to build a robust model to predict formation energy from materials composition with accuracy comparable to DFT-computations themselves (no structure information was considered though)^23^. Structure information is critical in performing DFT-computations and further experiments for validation; lack of structure information in predictive modeling results in limited applicability of such models in materials screening. Therefore, even though such composition-only based predictive models helps screen and identify potential material candidate without knowledge of geometry, we may observe significant prediction error with respect to ground truth for them, as they cannot distinguish between structure polymorphs, resulting in inaccurate screening for further time-consuming DFT-computations and expensive laboratory experiments during the process of materials discovery and design^25,34,44^. Another predictive modeling approach for formation energy of materials is by incorporating the crystal structure in model input by training them on DFT-computed datasets^43,49,50,64^. Nevertheless, a critical issue with DFT-computations based predictive modeling approach is that since DFT-computations used in the big materials datasets containing 1000s of inorganic compounds, which are generally used for materials property predictive modeling, have significant discrepancy against experimentally observed values; the predictive models based on them automatically inherit such discrepancy of DFT-computations from the training data. Consequently, the predictive models trained using DFT-computations automatically inherit such discrepancy, in addition to the prediction error with respect to DFT-computations themselves used for training; the discrepancy between DFT-computation and experiments serves as the lower bound of the prediction errors that can be achieved by the ML models with respect to experiments.

In this work, we demonstrate how AI can be used together with large DFT-computed datasets and existing experimental observations to build predictive models which can compute materials property more accurately than DFT by focusing on the critical materials science task of predicting “formation energy of a material given its structure and composition” using datasets that consists of inorganic compounds. One of the most elegant advantage of deep learning (AI) is the ability of perform transfer learning from large datasets to smaller datasets across similar domains^65^. It allows us to first train a deep neural network (DNN) model on a source domain with a large available dataset and then, fine-tune the model parameters by training again on the target domain with a relatively smaller available dataset. There exists multiple applications of deep transfer learning in scientific domains, from computer vision to computer networks, natural language processing, reinforcement learning to materials science and other scientific domains^23,66–70^. Here, we leverage deep transfer learning from DFT-computations with a simple deep neural network (DNN) model architecture - IRNet^42,53^, since it has been shown to outperform traditional ML algorithms without any need for domain knowledge based model architecture engineering; we do not explore other DNN models since they can be easily applied to our prediction task given the availability of experimental data in the required model input format. Note that there exist multiple DNN modeling approaches for predicting formation energy from materials structure using DFT-computed datasets^43,49,50,64^; our goal is illustrate how AI can compute formation energy from structure with better accuracy, rather than building a new DNN architecture for predictive modeling. We first train IRNet on the large DFT-computed dataset and then, fine-tune this model on the available experimental observations containing formation energy and materials structure information. When the model is trained on the large DFT-computed dataset, it learns a rich set of domain-specific features from the materials structure and composition provided in the model input, which proves critical in capturing the features present in the smaller (but more accurate) experimental observations to make accurate predictions for formation energy. On an experimental hold-out test set containing 137 entries, the AI model, trained on the large DFT-computations along with experimental observations by leveraging deep transfer learning, can predict the formation energy from materials structure and composition with a mean absolute error (MAE) of 0.064 eV/atom, which is significantly better when compared against DFT-computations ($>0.076$ eV/atom) for the same set of compounds. We believe our AI methodology for materials property prediction will play a complementary role to theoretical DFT-computations and help us in moving closer to experimental level prediction accuracy.

## Results

### Datasets

We have used three DFT-computed datasets and an experimental dataset in this study. The three DFT-computed datasets are: the Open Quantum Materials Database (OQMD)^5,6^, Materials Project (MP)^8^, and Joint Automated Repository for Various Integrated Simulations (JARVIS)^9,13–15^. The dataset from OQMD is composed of DFT-computed formation energy along with other materials properties. The dataset from MP is composed of inorganic compounds with a set of materials properties including formation energy. The experimental dataset (EXP) used in this study comes from the “exp-formation-enthalpy” dataset of Matminer (an open source materials data mining toolkit)^24^. All evaluations using DFT-computed datasets use a hold out test set using a random train:test split of 9:1; the evaluations using experimental datasets use a hold out test set such that entries from this test set is not contained in any training set in our study.

### Structure-based predictive modeling

A given crystalline material can be uniquely represented and identified using its composition and crystal structure. If we can predict the formation energy of a given material from its composition and structure with high accuracy, it can significantly reduce the further time, energy and cost associated with design and discovery of new materials for scientific and real world applications. In this section, we present an elegant approach to build a highly accurate predictive model for formation energy given material composition and structure using IRNet^42,53^ coupled with transfer learning. IRNet is a general purpose deep neural network that enables learning of materials properties in the presence of big materials datasets for accelerating materials discovery. IRNet take materials composition and structure in the form of numeric vector as inputs and predict formation energy as a regression output. IRNet takes 126 structure-derived attributes using Voronoi tessellations^43^ along with 145 composition-derived physical attributes^34^. We train IRNet on different DFT-computed datasets and on the experimental dataset; IRNet is trained with and without transfer learning from DFT-computed datasets on the experimental dataset. The mean absolute error (MAE) in predictions on the corresponding DFT and experimental (EXP) test sets are presented in Table 1.

**Table 1:** Performance of IRNet (AI) in predicting formation energy from material structure.

Model name	Model for TL	Training data			DFT test set		EXP test set	
		Name	Training set size	Validation set size	Size	MAE (eV/atom)	Size	MAE (eV/atom)
IRNet-JARVIS	–	JARVIS	20388	2224	3063	0.144	137	0.147
IRNet-MP	–	MP	101716	11224	12471	0.097		0.097
IRNet-OQMD	–	OQMD	352711	39142	43448	0.042		0.120
IRNet-EXP	–	EXP	522	28	NA	NA		0.327
IRNet-JARVIS-EXP	IRNet-JARVIS							0.087
IRNet-MP-EXP	IRNet-MP							0.078
IRNet-OQMD-EXP	IRNet-OQMD							**0.064**

Least MAE value on EXP test set is in bold.

From our modeling experiments, we observe that IRNet models trained by leveraging transfer learning from DFT-computations demonstrate significantly lower prediction errors compared to the IRNet models trained from scratch. Although the prediction error on DFT test sets can be quite small in the case of large datasets such as OQMD (0.042 eV/atom), the true prediction error against experimental values is significantly high (0.120 eV/atom). This happens because of the inherent discrepancy in model predictions which is learned from the DFT-computations used in training the model. For smaller datasets like JARVIS and MP, the model has high prediction error against DFT themselves since the learning capacity of the model is not saturated due to lack of training samples. When IRNet is trained from scratch solely using the existing small experimental observations (EXP), the performance is quite poor (0.327 eV/atom) as expected. There are only 522 training samples in this case, which is extremely small for a large model like IRNet with 17-layer deep neural network architecture. However, if we first train the model using a DFT datasets with sufficient training samples, the model learns to capture the approximate formation energy values for a given material structure and composition. In this study, we experimented with using a pretrained model from each of the three DFT datasets and fine-tuning them on the small set of experimental observations containing materials structure and composition. For the same fine-tuning dataset (EXP), the performance of the predictive model is directly related to the size of the DFT dataset used for pretraining. The best MAE of IRNet against experimental observations is achieved when we leverage the pretrained model from OQMD dataset. Here, we achieve an MAE of 0.064 eV/atom in predicting formation energy from materials structure and composition; this is the best result to our knowledge for this critical predictive task in materials science on experimental data.

### Discrepancy of DFT-computations

Next, we analyze how accurate the predictions using the proposed approach are as compared to existing DFT computations. We perform this study by analyzing the formation energy from DFT computations in the three datasets against the experimentally observed values, and comparing the IRNet predictions against the experimental observations as well. First we look into how the DFT-computations compare against experimental observations by looking at the common entries in the whole dataset. When analyzing IRNet predictions against experimental observations, we only leverage entries from the hold-out experimental test set since the entries in the training set are used for training the model.

**Figure 1:**
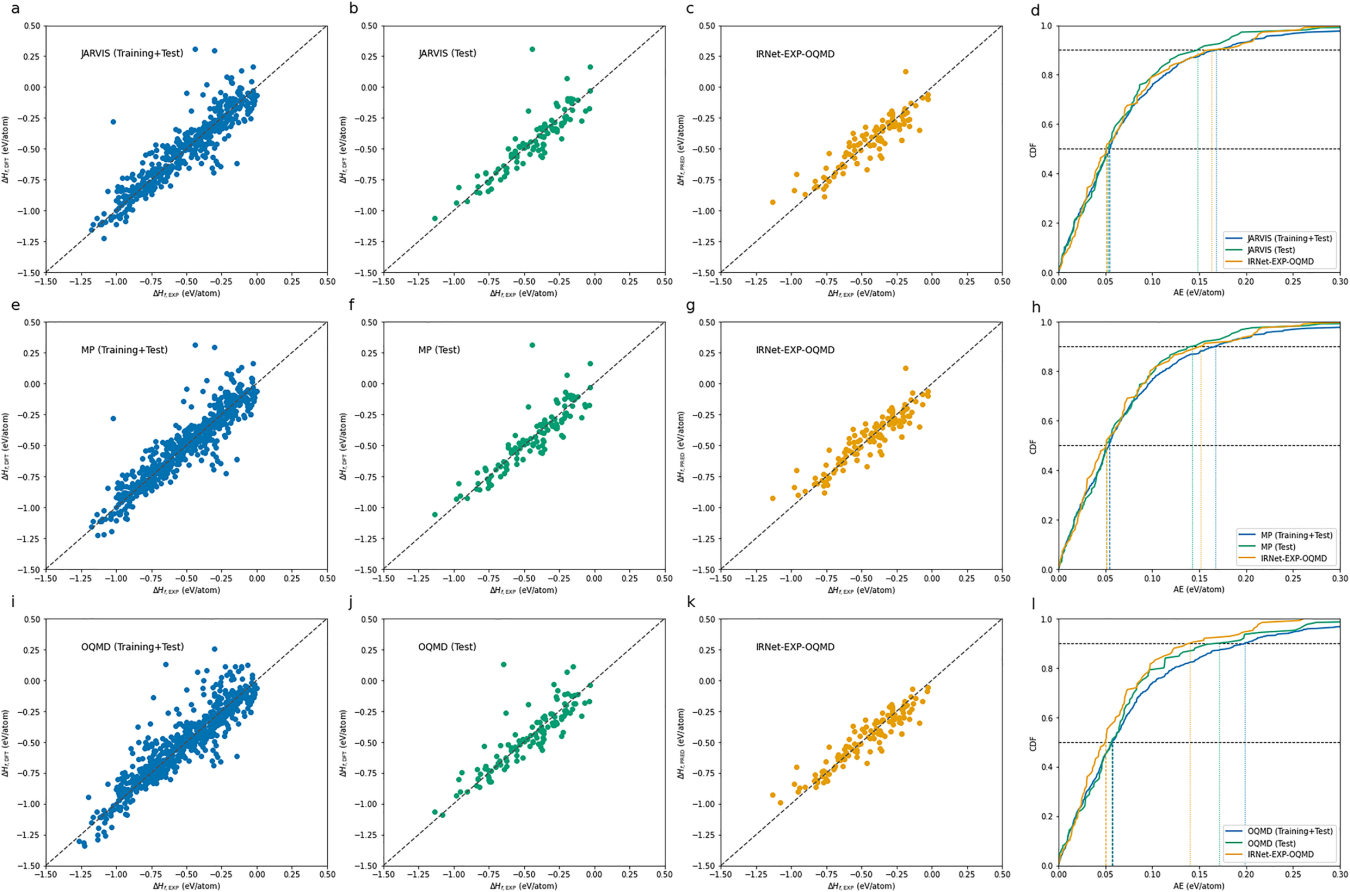
Comparison of DFT andIRNet (AI) predictions against experimental observations. The three rows represent the three DFT datasets-JARVIS (a-d), Materials Project (MP) (e-h), and OQMD (i-l); first column subplots (a, e, i) illustrate the formation energy from DFT database on y-axis vs experimentally observed values from EXP dataset on x-axis of the common compounds (training+test sets) between the DFT-computed dataset and experimental observations (EXP) for the three datasets; second column subplots (b, f, j) illustrate the formation energy from DFT database on y-axis vs experimental observed values on x-axis for the common compounds between DFT-computed dataset and the test set of EXP for the three datasets; third column subplots (c, g, k) demonstrate the IRNet predictions for the common compounds between DFT database and the test set of EXP (same entries as in the second column). The last column subplots (d, h, l) display the cumulative distribution function (CDF) of the DFT-computation error and IRNet prediction error against EXP dataset for the different set of common entries with EXP dataset that are displayed in the first three columns.

Figure 1 and Table 2 demonstrate the discrepancy of DFT-computations and our prediction model against the true experimental observations. We enlist the individual discrepancies of *all* the common entries (training+test sets) as well as the common entries present in *just* the test set for each DFT dataset due to different overlaps. From the scatter plots of DFT-computed formation energy vs the experimentally observed ones, we can clearly observe how significant the discrepancy can be for the individual entries as well as the overall datasets. The scatter plot distribution of the common entries from the test sets of different DFT-computed datasets follow similar trend to the common entries from the whole DFT-datasets. The median discrepancies in the DFT-computation for the test set agree with that of all entries from the same DFT-computation. The mean discrepancies for the test set seems to be better than the whole dataset signalling the test set entries are more closer to the experimentally observed values compared to other common entries with the DFT-computed datasets (also clear from the absence of entries with worst discrepancies in the scatter plot). The mean discrepancies for all entries are greater than 0.076 eV/atom for all three datasets; the error exceeding 0.167 eV/atom for the entries with worst (90th percentile in the CDF) DFT-computation discrepancy are as illustrated in the scatter plots. The discrepancies in our analysis are highest for the QOMD dataset, which also contain significantly more entries and have larger overlap with the EXP dataset. Note that we are leveraging the material structure as well as composition information when making these comparisons; measured discrepancy from our analysis are significantly lower than ($>0.1$ eV/atom) reported in the existing studies based on material composition only^6,17,22,23^.

The scatter plots of formation energy predictions using IRNet-OQMD-EXP (a IRNet model built on OQMD from scratch and then fine-tuned on EXP) clearly demonstrates how these predictions are closer to the experimentally observed values compared to the DFT-computations. The IRNet deep neural network is able to learn the mapping from the structure and composition of a given material to its formation energy better than the ones computed theoretically using different DFT computations in all three datasets. There is significantly lower discrepancy between the predicted formation energy against experimental observations as seen from the absence of noise in the scatter plots. The mean and median discrepancies in the predictions using IRNet-OQMD-EXP are lower than ones in DFT-computations for all three datasets. This is especially true for QOMD - the mean discrepancy of DFT-computations and predictions from IRNet-OQMD-EXP against experimentally observed values for the test set containing 125 entries are 0.078 eV/atom and 0.065 eV/atom ($20\%$ better). This demonstrates how DFT-computations can be leveraged together with available experimental observations using deep neural networks to build prediction models that can compute materials property more accurately using AI than DFT.

**Table 2:** Comparison of DFT and IRNet (AI) predictions against experimental observations.

DFT dataset	Training + Test sets		Test set		
	Size	DFT vs. EXP	Size	DFT vs. EXP	IRNet vs EXP
		MAE (eV/atom)		MAE (eV/atom)	MAE (eV/atom)
JARVIS	497	0.077	108	0.072	**0.071**
MP	607	0.076	126	0.071	**0.068**
OQMD	648	0.084	125	0.078	**0.065**

Least MAE values are in bold.

## Discussion

In this work, we have demonstrated how one can predict (compute) a materials property more accurately using AI than DFT by leveraging together existing collections of experimentally measure values and DFT-computations using a deep neural network. On an experimental hold out test set of 137 entries, IRNet (AI) can predict the formation energy from materials composition and structure with an MAE of 0.064 eV/atom against experimental observations; this is significantly better than the existing discrepancy in DFT-computations for the same set of compounds. Our analysis from comparing the common entries between DFT-computations and experimental observations (EXP) demonstrates that the discrepancy is $>0.076$ eV/atom; for the common entries in the hold-out test set from EXP and DFT-computed datasets, predictions using AI (IRNet-OQMD-EXP) are generally closer to experimentally observed values than using DFT. Current works in predicting formation energy using materials structure have mainly focused on designing deep neural network based models trained and tested using DFT-computations; in this work we put our focus away from building better deep neural network for the prediction task. Rather, we have leveraged the simple and elegant IRNet model that takes vector inputs and have been shown in outperform traditional ML models for materials property prediction from structure and composition without the need for any domain based model architecture engineering^42,53^; we believe other existing model architectures can be easily leveraged to take advantage of our proposed approach of transfer learning for better performance. Here, we are using both materials structure and composition for our experiments and analysis. Previous studies have explored the discrepancy of DFT-computations against experimentally observed values on different sets using material composition only; they generally compare the lowest DFT-computed formation energy (which represent the most stable structure for a given material composition), and reported the discrepancy to be $>0.1$ eV/atom in all cases; the measured discrepancy from our analysis are significantly lower than the ones reported in the existing studies based on material composition^6,17,22,23^. Kirklin et al.^6^ reports the MAE of the experimental values to be 0.082 eV/atom, based on a comparison between 75 compounds with same composition common to two experimental data sources used in his study; however, these sources did not contain the structure information and this comparison was based on lowest formation energy for a given composition (the most stable structure for a given material composition). We performed a similar comparison between our experimental dataset (EXP) and SSUB^6,23^ based on composition only; the MAE between the two experimental datasets is 0.054 eV/atom for the 67 entries (maximum common entries) with common composition, without considering materials structure information. The new insight gained from our study provide a more accurate way to look at previous research works focused on comparison between DFT-computed formation energy and experimentally observed ones; they also provide a new way to investigate the existing research works geared towards accurate prediction modeling based on DFT-computations. Note that while DFT-computations are still critical to understand the chemical and physical properties of crystalline materials, this research demonstrates how they can be leveraged together with existing experimental observations to build robust AI-based predictive models that can predict materials properties with better accuracy than the DFT-computations themselves. Such AI models can provide a faster and more accurate way to perform rapid screening for potential materials candidates to reduce the composition-structure space for further experiments for accelerating materials discovery and design. Since AI models can be built to compute/predict materials property more accurately than DFT, this might significantly reduce the need for carrying out time-consuming (takes around 10 hours for a single input even on a supercomputer) DFT-computations in the future. Note that AI models are trained using the large DFT-computed datasets and existing experimental observations; hence, the AI methodology presented in this work is a complement to DFT-computations for moving closer to experimental level prediction accuracy by quickly screening a large number of compounds using the fast AI models, and performing the slow DFT calculations for only the most promising compounds. Our work demonstrates the advantage of leveraging existing small, but ground truth data (experimental observations) along with computational/simulation data for moving closer to more accurate and robust predictions. We believe this could open up a new research direction for prediction modeling, not only for the critical problem of prediction of formation energy from materials structure and composition and other properties in materials science, but also for other materials properties (e.g. band gap), other materials classes (e.g. polymers), and even other scientific domains (e.g. climate science), where the high-fidelity ground truth is scarce and hard to obtain, but low-fidelity simulation/computations data exist in plenty.

## Methods

### Data cleaning and preprocessing

We have used three DFT-computed datasets and one experimental dataset in this study. The three DFT-computed datasets are: the Open Quantum Materials Database (OQMD)^5,6^, Materials Project (MP)^8^, and Joint Automated Repository for Various Integrated Simulations (JARVIS)^9,13–15^. The dataset from OQMD is composed of DFT-computed materials properties comprising of formation enthalpy and other materials properties such as band gap, energy per atom, and volume. The dataset from MP is composed of inorganic compounds with their formation energy along with some other materials properties such as band gap, density, energy above hull, energy per atom, magnetization and volume. The experimental dataset (EXP) used in this study comes from the “exp-formation-enthalpy” dataset of Matminer (an open source materials data mining toolkit)^24^. Matminer is a Python library that contains a collection of routines for obtaining data on materials properties from various databases such as Materials Project and OQMD, featurizing the complex materials attributes such as composition, crystal structure and band structure into physically-relevant numerical quantities for building machine learning based prediction models, and also the tools for analyzing the results from data mining. We obtain the material structure information for each entries by mapping the ’oqmd_id’ and ’mp_id’ to our OQMD and MP datasets respectively; if the ’oqmd_id’ is not present, ’mp_id’ is used instead; we discard the entries without any of these two columns. We drop all the entries with any missing or NaN values. Also, the entries with formation energy outside the range of $-20$ eV/atom to 5 eV/atom are dropped in all datasets. All evaluations using DFT-computed datasets use a hold out test set using a random train:test split of 9:1; the evaluations using experimental datasets use a hold out test set such that entries from this test set is not contained in any training set in our study.

### Models and tools

We have used the IRNet^42,53^ architecture with composition-structure as model inputs in this study. They are implemented and trained using Python and TensorFlow^71^ framework. The model inputs for IRNet are composed of a set of 145 composition-derived physical attributes^34^ and 126 structure-derived attributes^43^. Models trained using DFT-computed datasets are used as pretrained models for transfer learning on experimental dataset.

## Data Availability

No datasets were generated during current study. All the datasets used in the current study are available from their corresponding public repositories- OQMD (http://oqmd.org), Materials Project (https://materialsproject.org), JARVIS (https://jarvis.nist.gov), and experimental observations (https://github.com/wolverton-research-group/qmpy/blob/master/qmpy/data/thermodata/ssub.dat).

## Code availability

All the codes required to train the IRNet model used in this study is available at https://github.com/dipendra009/IRNet.
